# Variation of shell ornamentation with latitude and water depth—A case study using living brachiopods

**DOI:** 10.1002/ece3.10006

**Published:** 2023-04-21

**Authors:** Facheng Ye, Maria Aleksandra Bitner, Guang Rong Shi

**Affiliations:** ^1^ School of Earth, Atmospheric and Life Sciences, Faculty of Science, Medicine and Health University of Wollongong Wollongong New South Wales Australia; ^2^ Institute of Paleobiology, Polish Academy of Sciences Warszawa Poland

**Keywords:** bathymetry, brachiopods, latitude, macroevolution, ornamentation index, shell ornamentation, shell predation

## Abstract

As a potential anti‐predatory defensive structure, the shell ornamentation of marine calcifiers is usually used to understand the macro coevolution of the interactions between predators and preys. Marine calcifiers' shell ornamentation complexity is generally believed to vary negatively with latitude and water depth. In this paper, we explored the association between shell ornamentation and latitude/bathymetry using the latest global database of living brachiopods. We found that (1) ~59% of living brachiopods species are characterized by smooth shells and that (2) there is no statistically significant linear trend, either positive or negative, between the ornamentation index and latitudes nor with water depths. Both findings are puzzling for living brachiopods as they are sharply contrasted to the patterns of fossil brachiopods whereby the latter, especially Paleozoic brachiopods, are known to exhibit (1) a much greater ornamentation diversity and (2) (at least for the geological periods that have been studied) a linear latitudinal gradient of ornamentation complexity existed. The reasons why living brachiopods have such a high proportion of smooth or weakly ornamented shells and fail to demonstrate an unequivocal linear latitudinal ornamentation gradient were explored and are linked to a multitude of potential factors rather than uniquely only to the predation pressure. Among these, the most plausible factor seems to be the cryptic (refuge‐type) habitats (e.g., deep waters, cold polar regions, and submarine rock caves) that living brachiopods have been adapted to due to their low metabolism, where predation pressure is low, allowing brachiopods to enact the predator avoidance strategy rather than having to manufacture robust shell ornamentation to survive in an otherwise highly engaged predator–prey global marine ecosystem.

## Graphical abstract

Through used the latest database, the shell ornamentation of living brachiopods has changed markedly compared with their fossil counterparts, growing with a much higher proportion of smooth shells. However, the association between the latitude and shell ornamentation is not simply linear correlated as straightforward as anticipated. This result may reflect that predation pressure is not the unique driver of the diversification of shell ornaments of living brachiopods.

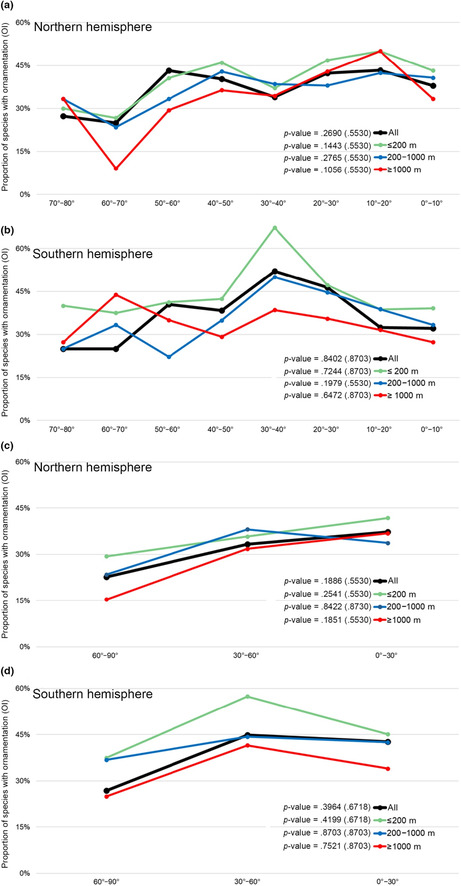

## 1 INTRODUCTION

External hard skeletons of marine calcifiers are known to serve multiple functions (Freestone et al., 2011; Harper, 2006; Immenhauser et al., 2016; Wood, 2018). Among them, protection against predation is a significant driver for diversifying their mineralized skeletons (Klompmaker & Kelley, 2015; Vermeij, 1977, 1989). A body of previous research has noted an apparent association between shell ornamentation (e.g., spines, costae, rugae) and external predation pressure irrespective of living or fossil organisms, leading to a general conclusion that shell ornamentation may be considered to represent a highly effective anti‐predatory defense mechanism (Leighton, 1999; Nicol, 1967; Vörös, 2005; Ward, 1981; Wu et al., 2019). Such a general paradigm, the so‐called “arms race” or the escalation theory (Vermeij, 1987, 1994, 2002), might have started as early as in the Precambrian and is also broadly accepted as one of the plausible evolutionary triggers for both the Cambrian biodiversity explosion (e.g., Bengtson, 2002; Wood & Zhuravlev, 2012; Zhang et al., 2014), and the Mesozoic Marine Evolution (e.g., Vermeij, 1977, 1987).

However, aside from serving as a plausible anti‐predatory defense means, shell ornamentation of marine organisms may also support other important ecological functions, either partially or solely. For example, it has been shown that reducing ornamentation can generally help bivalves burrow faster than ornamented species (Alexander et al., 1993; Eagar, 1978). And, at least for fossil brachiopods, dense, long, and slender body spines were thought by some to serve as anchors to prevent living brachiopods from sinking into and being buried within muddy substrates (Grant, 1968; Rudwick, 1970; Thayer, 1975). Other known examples of shell ornamentation in facilitating or enhancing certain specialized ecological functionalities include increased buoyance and movement in aquatic environments (e.g., Alexander, 1984; Chamberlain & Westermann, 1976; Hornbach et al., 2010; Tabita Symphonia & Senthil Nathan, 2021), morphological plasticity to cope with the environmental crises during mass extinction intervals (Dai et al., 2021; Dal Corso et al., 2022), increased shell surface area to volume ratio as an adaptation to deoxygenated habitats in post‐mass extinction ecosystem (Wu et al., 2019), and sexual dimorphism (Klug et al., 2015; Schilthuizen, 2003).

Brachiopods are marine sessile benthic suspension feeders, and their two calcified valves (ventral valve and dorsal valve, respectively) protect their soft parts from predators (James et al., 1992; Williams, 1997). Due to their relative great abundance and global distribution in the Paleozoic marine fossil record, fossil brachiopods have commonly been used as effective ecological indicators for reconstructing paleo‐environments (Angiolini et al., 2007, 2009; Brand et al., 2011; Curry & Fallick, 2002; Shi et al., 1995). For the same reason, compounded by their sessile lifestyle, brachiopods are vulnerable as the prey of a range of marine predators, either drillers or durophagous (Alexander, 1981, 1990; Emig, 1997; James et al., 1992; Leighton, 2003; Peck, 1993, 2001; Richardson, 1997; Witman & Cooper, 1983). Fossil brachiopods have been considered among the ideal organisms for studying the dynamic predator–prey interactions and their spatial–temporal trends through geological time (Rudwick, 1970; Signor & Brett, 1984; Table 1). One of the major conclusions of these studies is the revelation that the overall strength and complexity of fossil brachiopod shell ornamentation appears to bear strongly on latitudes in that they tend to become more complicated, more frequent, and stronger toward lower latitudes or warmer environments, an apparent association that has been linked to the latitude‐related predation pressure gradient (Dietl & Kelley, 2001; Leighton, 1999; Wu et al., 2019).

**TABLE 1:** Summary of key studies documenting the brachiopod ornament characteristics at different latitudes/environments/ages.

Analyzed taxa and age	Classification of ornamentation	Remarks	Reference
Strophomenides: Devonian	Four categories	There is a negative correlation between shell ornament and latitude	Leighton (1999)
Articulates: Carboniferous	Four categories	Latitudinal gradient of shell ornament can be found in Carboniferous, and the gradient was more significant in Tournaisian, when the temperature gradient is steeper	Dietl and Kelley (2001)
Productida: Permian	Four classes	The proportion of different types of shell ornament fluctuates in different periods of Permian	Zhang and He (2008)
Rhynchonellides and terebratulides: Mesozoic	Four categories	Shell ornament steadily increases through the Triassic and Jurassic, then a sudden decrease happens during the latest Jurassic and Early Cretaceous	Vörös (2010)
All groups of Brachiopoda: Jurassic	Four categories	The strongest shell ornament occurs in the high paleolatitudes (45° N); decreases in the mid‐latitudes and reaches a high value again near the equator	Vörös (2014)
All groups of Brachiopoda: Late Permian	Four types	There is an inversely correlated latitude‐ornament gradient	Wu et al. (2019)

Compared with fossil brachiopods, the spatial and environmental variations of shell ornamentation of living brachiopods have been little studied, with possibly only two exceptions. Thayer (1975) examined a range of brachiopod external morphological characters, including shell size, shape, and ornamentation, to determine their relative significance in aiding the brachiopods living in soft bottom substrates. On the contrary, Alexander (1990) used experiments to investigate and compare the relative strength of costate, spinose, and lamellose shells of selected living brachiopod shells in resisting shell‐crushing forces from predators. To our knowledge, to date, no study has been undertaken to explore the variation of ornamentation in living brachiopods in relation to latitude or water depth.

Using the latest global database of living brachiopods recently established by Ye et al. (2021), this paper sought to provide a novel study of how the surface ornament of living brachiopod shells varies with latitude and bathymetry. In particular, the study aimed to test the hypothesis that the strength of shell ornament, as a proxy for an anti‐predatory defense mechanism against predation, decreases with latitude and water depth (Ashton et al., 2022; Freestone et al., 2021; Harper & Peck, 2016; Oji, 1996; Reynolds et al., 2018). In other words, if the scenario that intense predation pressure usually occurs in low latitudes and shallow water areas holds, we then should expect to see an increase of more brachiopods with ornamentation, and more complicated or stronger ornamentation to be more frequently associated with brachiopods living in lower latitude or shallower water habitats (or habitats near the continental shelf). Through these investigations, we aimed to elucidate the main potential controlling factors that govern the variation of shell ornamentation. As such, this study adds to an improved understanding of how species interact with predators or other biotic and abiotic variables in different latitudes and water depths, either in the geological past or in the modern world.

## 2 DATA ASSEMBLY AND METHOD

### 2.1 Database

For this study, an extended database with shell ornamentation‐enriched information was built based on an earlier version of the global database built for living brachiopods (Ye et al., 2021). In brief, in addition to the well‐defined basic information of living brachiopods (such as taxonomy, geo‐coordinated, and water depth distribution), various attributes of shell ornamentation of corresponding species have been added to the new database, sourced primarily from the published literature by referring to a range of information including text descriptions and illustrations (figures/plates of photographed brachiopod images). The identity and taxonomy of the species included were initially based on the classification on BrachNet (http://paleopolis.rediris.es/BrachNet/) and further verified by one of the authors (M.A.B.). The data acquisition procedures and the exclusion of doubtful records were described in more detail by Ye et al. (2021). Finally, a total of 14,312 geo‐referenced occurrences, including 342 species of living brachiopods with adequate ornamentation information (i.e., at least with ornamentation either described in the text or illustrated in the figures) in our final dataset, were accepted and used for further analysis.

### 2.2 Methodology

#### 2.2.1 Shell ornamentation from different latitudes and water depth

**FIGURE 1:**
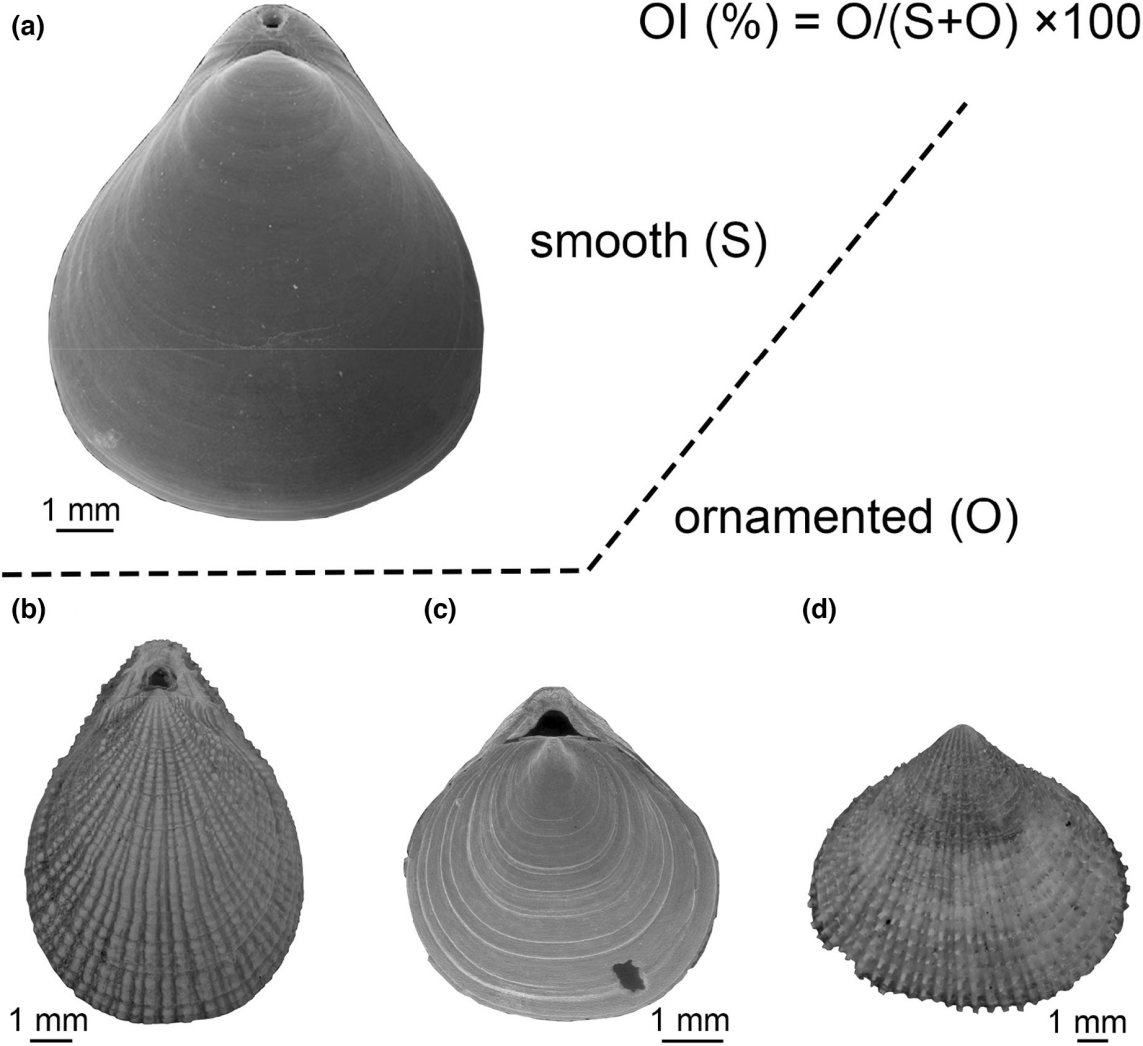
Illustration of the ornamentation categories used for living brachiopods. (a) Smooth shells or shells characterized with very weak ornaments (marked with only growth lines), *Xenobrochus rotundus* Bitner, 2008 (from Bitner, 2019); (b) radial ornamentation (e.g., ribs, costae), *Terebratulina japonica* (Sowerby, 1846) (from Bitner & Romanin, 2018); (c) concentric ornamentation (e.g., lamellose, rugose), *Acrobelesia cooperi* (d'Hondt, 1976) (from Simon et al., 2016); (d) spine & others, *Acanthobasiliola doederleini* (Davidson, 1886) (from Bitner & Romanin, 2018).

New technology like 3D measurement undoubtedly can provide more comprehensive data on shell ornamentation (e.g., Miao et al., 2022). However, owing to the limitations of inaccessible specimens and equipment, we adopted the methods of measuring and quantifying shell ornamentation that had successfully been applied to fossil brachiopods (Dietl & Kelley, 2001; Leighton, 1999; Vörös, 2010, 2014; Wu et al., 2019; Zhang & He, 2008; Table 1). In doing so, we first categorized living brachiopods into two elementary groups: smooth (*S*), which also included the species characterized by very weak ornaments (marked with only growth lines) and ornamented (*O*) (Figure 1). We then quantified the proportion of species with ornamentation inhabiting a geographic area (e.g., a latitudinal belt or bathymetric zone) as the Ornamentation Index (OI), namely OI (%) = *O*/(*S* + *O*) × 100 (Figure 1). To further investigate the manifestation of the types of ornament in relation to latitude and water depth, we subdivided ornamented brachiopods into four distinct types: species with radial ornament (RO), species with concentric ornament (CO), species with spine and others (SO), and species with multiple types (M) (Figure 1, Table 2). It should be noted that each species can only be classified into one single ornamentation type.

**TABLE 2:** Value matrix of standardized residuals (or Pearson residuals), indicating the strength of association (by using different colors) between different latitudinal bin versus ornamentation type. A larger positive value implies a stronger attraction between the corresponding row and column variable, and vice versa. It should be noticed that only the second and third columns are used for testing the latitudinal trend (see Figure 2a,b).

Latitude	Smooth (*S*), marked with only growth lines	Ornamented (*O*)	Ornamented (*O*)			
			Radial ornament (RO)	Concentric ornament (CO)	Spine and others (SO)	Multiple types (*M*)
70°–80° N	0.535	−0.658	−0.628	−0.674	−0.374	0.395
60°–70° N	0.852	−1.048	−0.694	−0.909	−0.504	−0.205
50°–60° N	−0.271	0.333	0.169	−1.24	0.773	1.147
40°–50° N	−0.054	0.066	0.246	−0.460	1.246	−0.548
30°–40° N	0.732	−0.900	−0.651	−1.001	−0.209	0.019
20°–30° N	−0.331	0.407	0.667	0.342	0.591	−0.943
10°–20° N	−0.576	0.709	0.877	0.498	−0.209	−0.391
0°–10° N	0.182	−0.224	0.151	0.392	−0.858	−0.827
10°–0° S	0.724	−0.890	−1.544	−0.803	−0.820	2.078
20°–10° S	0.790	−0.972	−0.505	−1.078	1.223	−1.064
30°–20° S	−0.838	1.031	1.068	0.498	−0.209	−0.019
40°–30° S	−1.542	1.897	1.638	0.569	−0.177	0.929
50°–40° S	0.145	−0.179	−0.231	0.333	0.273	−0.356
60°–50° S	−0.057	0.071	−0.542	1.722	−0.730	0.263
70°–60° S	0.934	−1.149	−1.836	1.014	−0.552	0.434
80°–70° S	0.762	−0.938	−1.186	0.418	−0.451	0.018

Standardized residuals:
3.001 to 6
1.501 to 3
1.001 to 1.5
0.501 to 1
0 to 0.5
−0.5 to 0
−1 to −0.501
−1.5 to −1.001
−3 to −1.501

Moreover, to quantitatively compare the differences in radial ornament (RO) between different latitudinal zones, we adopted a method devised by Wu et al. (2019) in quantifying the strength of radial ornaments (e.g., costae, ribs) by counting the number of costae crossing the maximum width of a specimen, then divided by the maximum width in millimeters (mm) of the same specimen. This index, called the Radial Ornamentation Index (ROI), was simply calculated as: ROI = *N*_radial ornaments at maximum width_/Width_maximum_ (mm). In most cases, holotypes or paratypes (20/29 of measured specimens are holotypes) were used for radial ornament measurement and quantification. Where neither the holotype nor paratype specimens were available, we chose one complete and fully illustrated adult specimen from the literature where unequivocal observations on its radial ornamentation could be determined.

**FIGURE 2:**
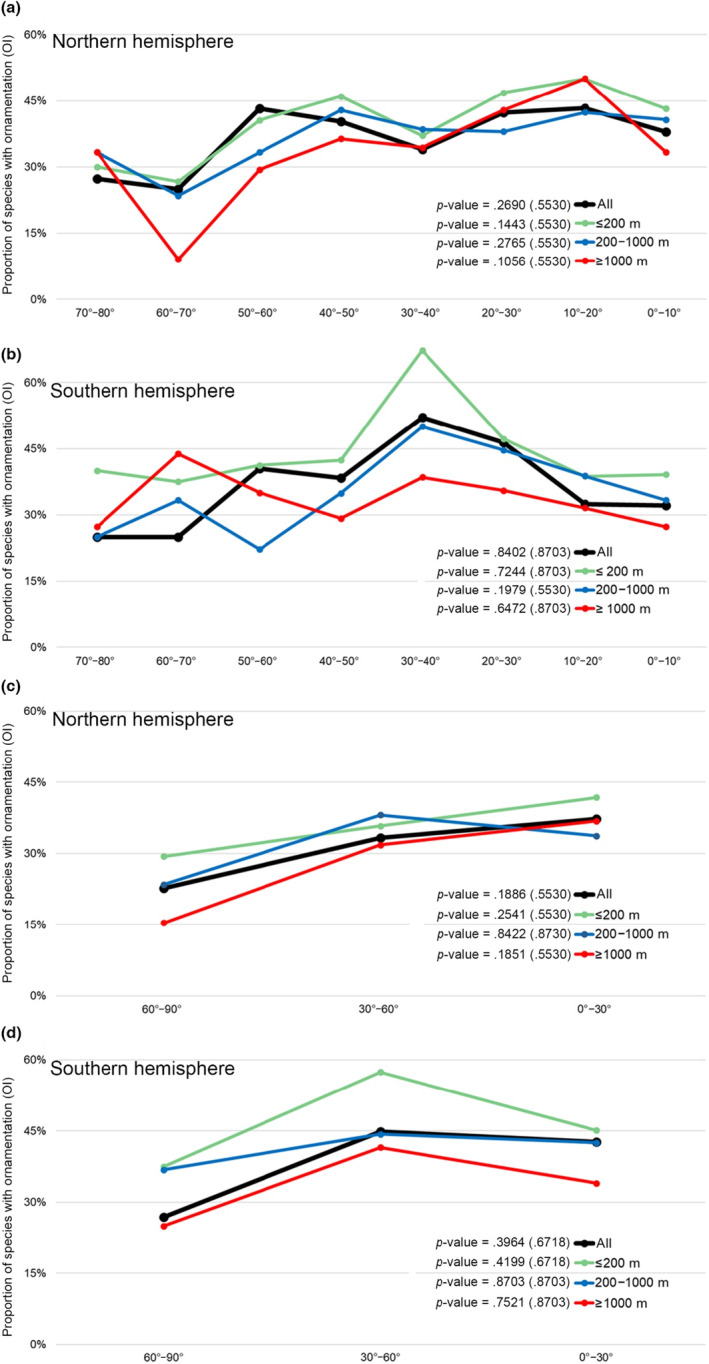
Variation of shell ornamentation index (expressed by OI) along the latitudinal gradient. (a) Northern hemisphere in 10° latitudinal bins; (b) Southern hemisphere in 10° latitudinal bins; (c) Northern hemisphere in 30° latitudinal bins; (d) Southern hemisphere in 30° latitudinal bins. Trend gradient and the corresponding *p*‐value were both tested by the Cochran–Armitage trend test, and the adjusted *p*‐values shown in parentheses.

**FIGURE 3:**
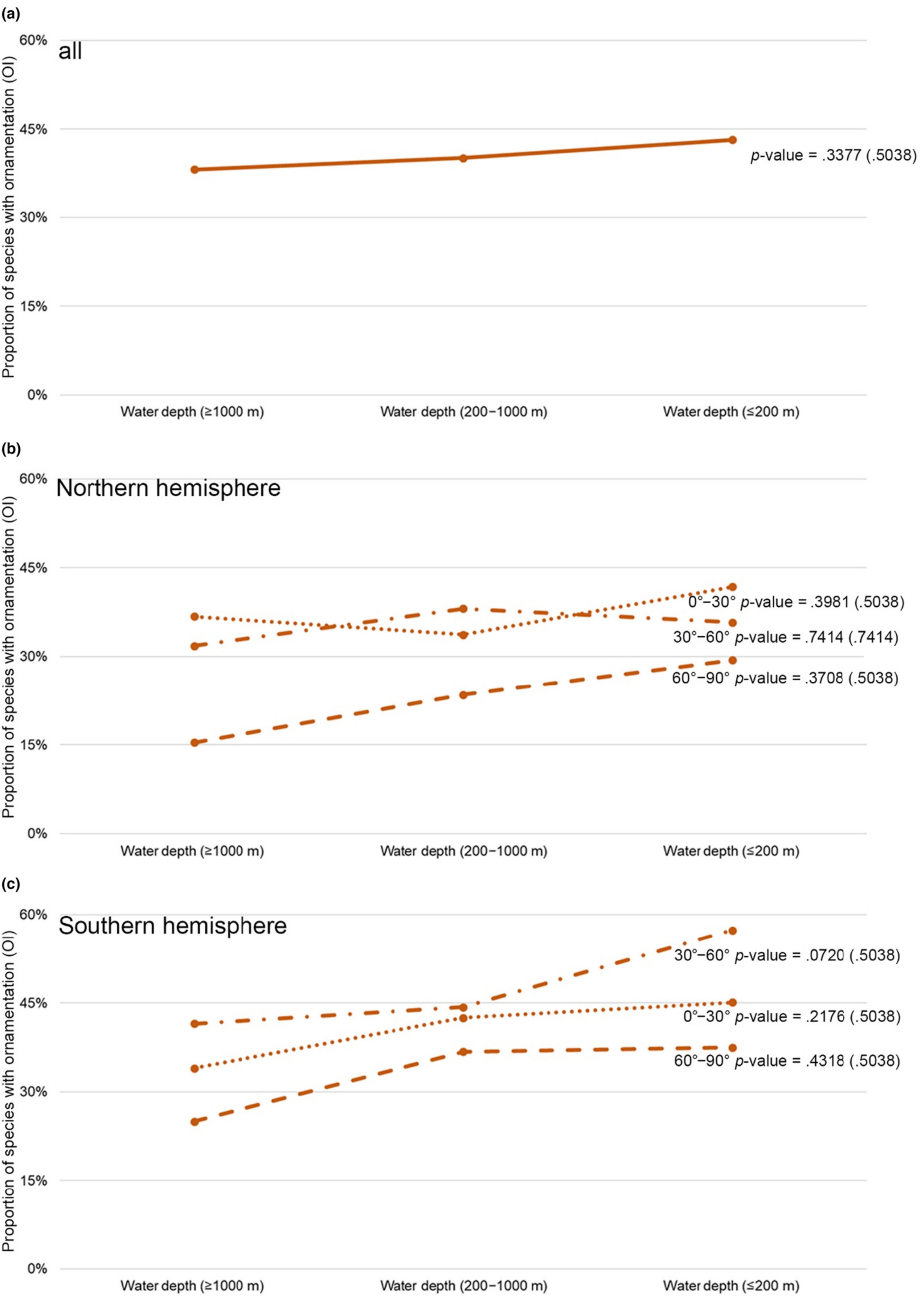
Variation of shell ornamentation index (expressed by OI) along the bathymetric gradient. (a) All data in three water depth intervals; (b) Northern hemisphere in three water depth intervals; (c) Southern hemisphere in three water depth intervals. Trend gradient and the corresponding *p*‐value were both tested by the Cochran–Armitage trend test, with adjusted *p*‐values shown in parentheses.

**FIGURE 4:**
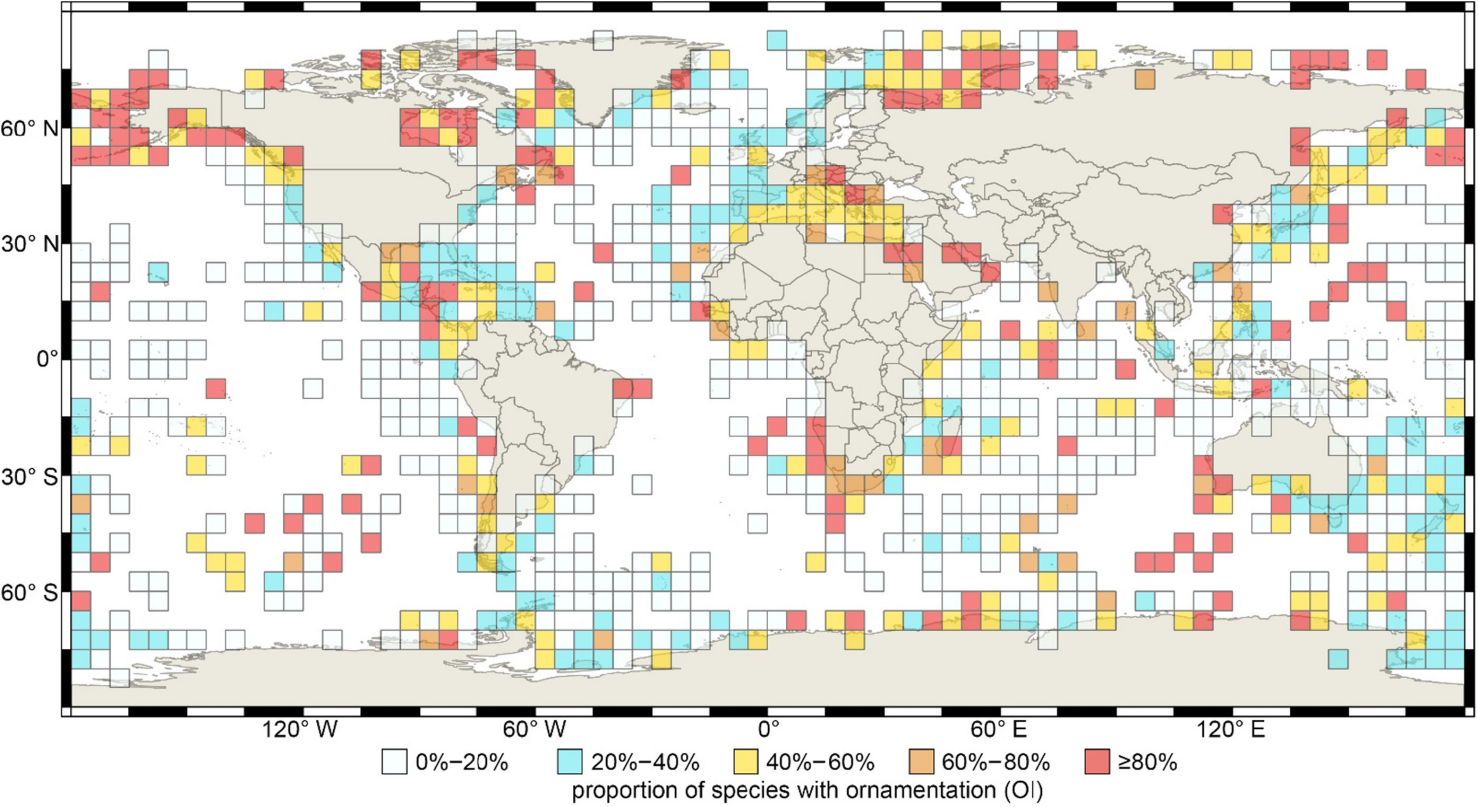
5° grid cell map of shell ornamentation index (expressed by OI) of living brachiopods. Different color represents the gradient of OI values as shown in the legend boxes. *Source*: global basic map was downloaded from ArcWorld Supplement via ESRI, then adapted for visualization here by using open‐source Geographic Information System QGIS (http://qgis.osgeo.org).

**FIGURE 5:**
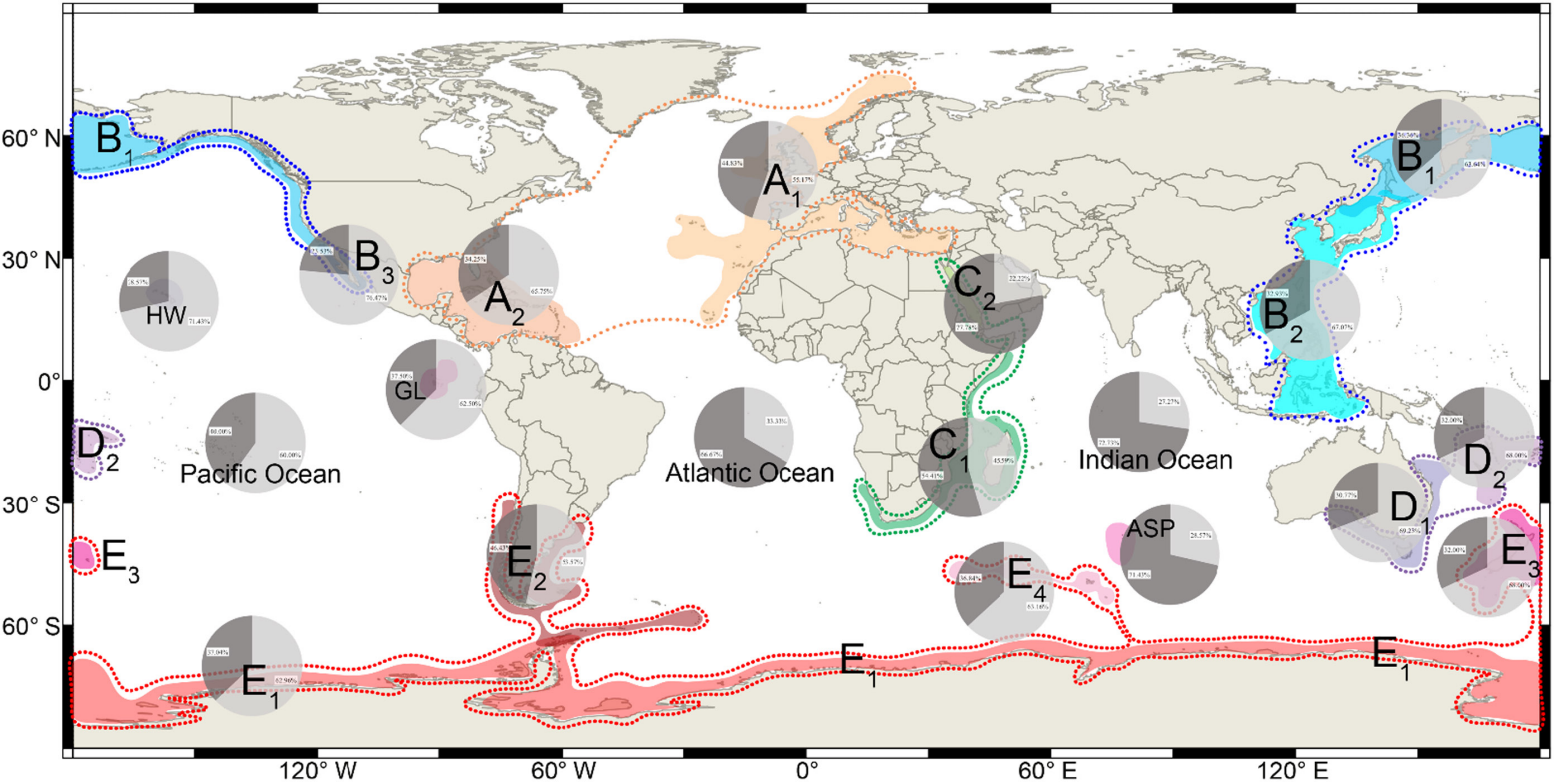
Global map showing the variation of shell ornamentation index (expressed by OI) from different bioprovinces (Ye et al., 2021). Different colors from the pie chart indicate the proportion of OI (dark gray: with ornamentation, light gray: smooth).

Next, we explored whether latitude and water depth contribute to living brachiopods' shell ornamentation, and parameterized certain attributes of the relevant latitudinal/bathymetrical variables for further statistical analysis. These parameters included: (1) two different latitudinal spatial scales, respectively at 10° and 30° wide intervals (Figure 2, Table 2); (2) different water depth intervals (≤200 m, 200–1000 m, and ≥ 1000 m, Figure 3), and (3) 5° grid cells of the global geographic map (Figure 4). The cutting‐off point between neighboring categories (latitudinal and bathymetric intervals) in each case was arbitrarily decided but still can be compared with other previous studies (Cox et al., 2019; Harper & Peck, 2016; Jablonski et al., 2006; Webb et al., 2010). In addition, we also tested OI of living brachiopods in relation to their bioprovinces as defined by Ye et al. (2021), plus the three additional open ocean bioprovinces: Atlantic Ocean, Indian Ocean, and Pacific Ocean (Figure 5).

#### 2.2.2 Statistical analyses

**FIGURE 6:**
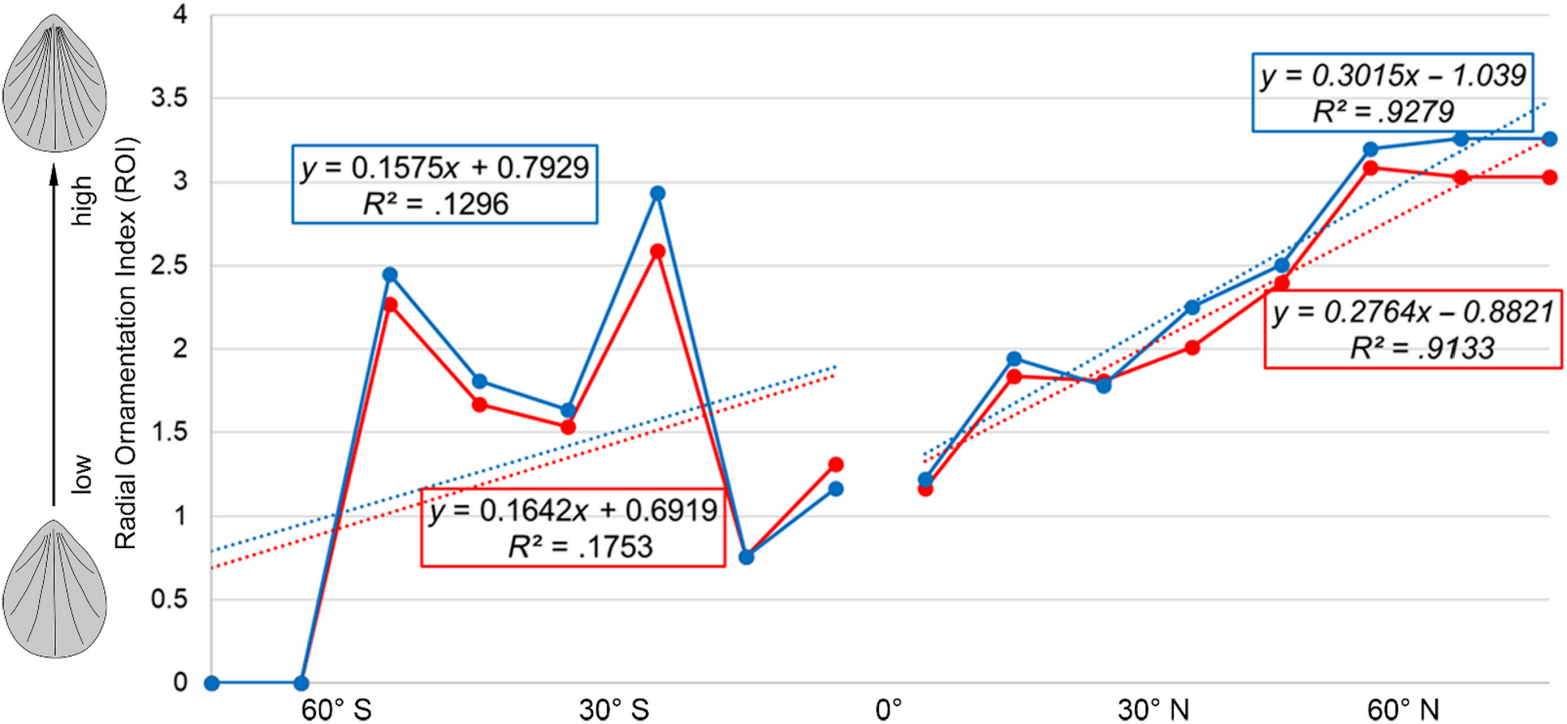
The changes of the ROI along different latitudinal zones, showing the higher ROI, the denser radial ornamentation. The data from ventral valve: blue color, the data from dorsal valve: red color. Equation and corresponding *R* ^2^ value in italic indicate *p*‐value ≤.05.

We applied the two‐tailed Cochran–Armitage trend test to assess whether there is any gradient trend of shell ornament (using “ornamented” and “smooth” categories as response variables) in relation to latitudes (Figure 2) and water depth (Figure 3). This trend test was usually used for binomial proportional data with a binary outcome (Salanti & Ulm, 2003; Mack et al., 2018, here, the binary results are (*S*) and (*O*)). In this study, the null hypothesis for the Cochran–Armitage test was that there is no trend of OI against a given category (each independent line in Figures 2, 3, and 6). In the statistical testing, a significance level or *p*‐value smaller than .05 was taken as strong evidence to reject the null hypothesis. In order to control the study‐wide type I errors that often occur when performing above multiple tests, *p*‐value adjustments were also applied for all the *p*‐values of trend tests through the Benjamini–Hochberg method (Thissen et al., 2002). In order to better visualize the structure of the association between five different types of ornament [(*S*), (RO), (CO), (SO), and (M)] under various latitudinal bins (Table 2), the original data matrices consisting of the frequency of the five particular types of ornament were converted into new data matrices containing standardized residuals derived from the chi‐square test. Such tests were often used for inspecting and decoupling the relationship between the row and column profiles (Franke et al., 2012; McHugh, 2013). We also calculated the chi‐square distance between the given latitudinal bin profile (frequency of the five types of ornament) and the average latitudinal bin profile (sum of the rows divides total marginal distribution) accordingly (Sourial et al., 2010; Table 3). Such a method is used to demonstrate if there is any gradient change of the dissimilarity along the latitudes (Wu et al., 2019). Linear regression analysis test was also performed on the distribution of the radial ornamentation type (RO; Figure 6). These tests were performed to verify the null hypothesis that there is no association between the radial ornamentation (ROI) and latitudes, with a *p*‐value smaller than .05 as evidence to reject the hypothesis. No such analyses were feasible for the other three ornamentation types (CO, SO, *M*) due to very small sample size (the three types of ornament combinedly only accounted for 12% of the total species included). Similarly, no such analysis could be done for the radial ornament type at different water depths due to the limited data availability.

**TABLE 3:** Chi‐square distances of ornamentation composition between each latitudinal bin and average profile, the larger value the higher dissimilarity, 0 means complete similarity.

Latitude	Chi‐square distances
80°–70° S	0.148
70°–60° S	0.240
60°–50° S	0.092
50°–40° S	0.006
40°–30° S	0.067
30°–20° S	0.022
20°–10° S	0.069
10°–0° S	0.161
0°–10° N	0.028
10°–20° N	0.016
20°–30° N	0.018
30°–40° N	0.021
40°–50° N	0.032
50°–60° N	0.096
60°–70° N	0.117
70°–80° N	0.130

**FIGURE 7:**
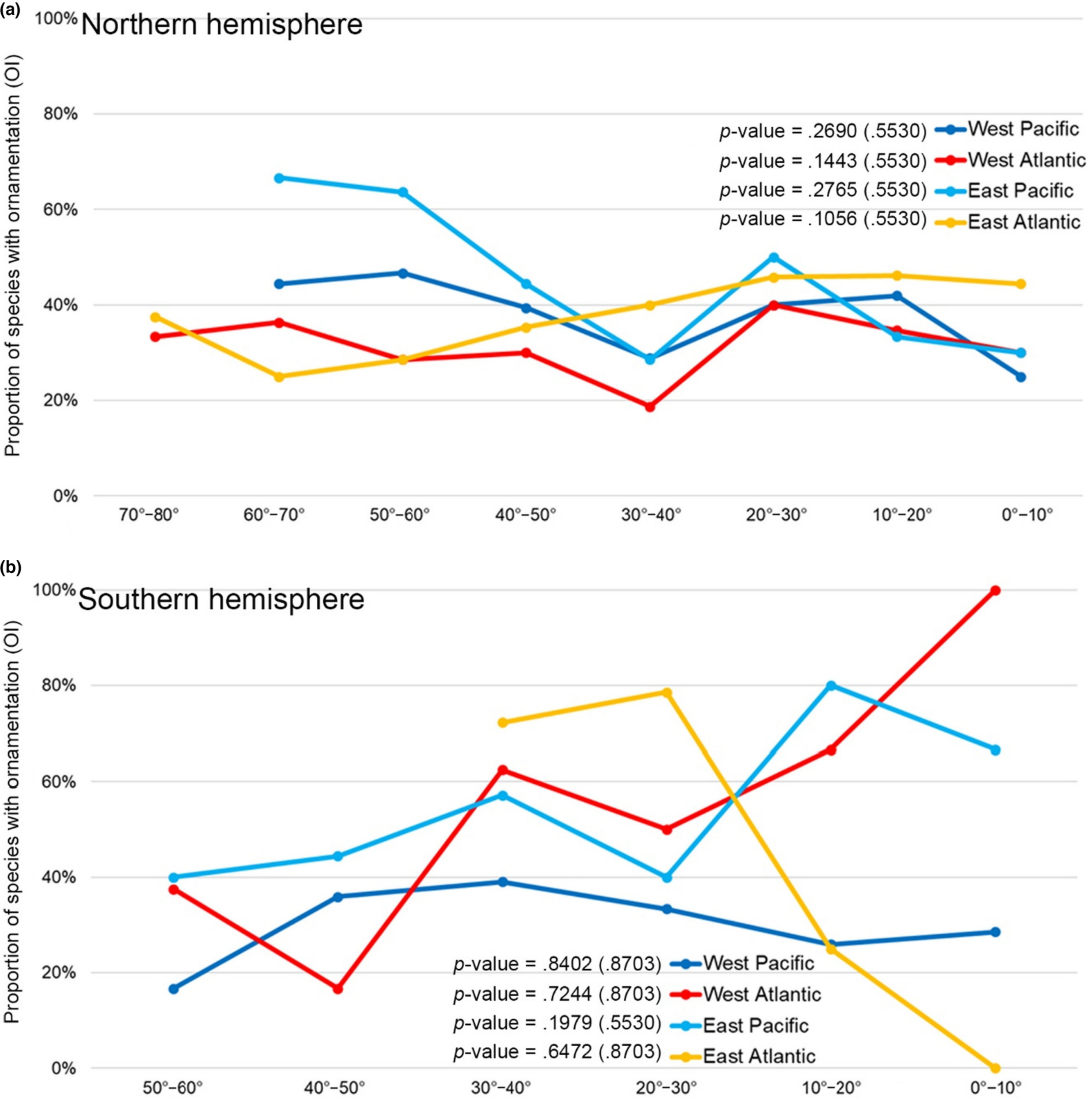
Variation of shell ornamentation index (expressed by OI) along the latitudinal gradient. (a) Northern hemisphere in 10° latitudinal bin from different coastlines; (b) Southern hemisphere in 10° latitudinal bin from different coastlines. Trend gradient and the corresponding *p*‐value were tested by the Cochran–Armitage trend test, with adjusted *p*‐values shown in parentheses.

All statistical analyses (Benjamini–Hochberg *p*‐value adjustment, Cochran–Armitage trend test, chi‐square test, and Pearson product–moment correlation coefficient test) were performed using R language Statistical Software (v4.1.3; R Core Team, 2022).

Lastly, we applied Spatial Autocorrelation Analysis (Moran's I; Moran, 1950) through ArcMap 10.8 (ESRI, 2011) to statistically investigate whether the values of OI are autocorrelated through space (using the data of 5° grid cells from Figure 4). Since the distance band parameter can affect the results of global Moran's calculation, we also used the incremental spatial autocorrelation tool from ArcMap to demonstrate the appropriate distance value where the spatial clustering becomes pronounced (Figure S1).

## 3 RESULTS

In summary, 59.1% of living brachiopod species are characterized by smooth shells. There is a considerable degree of variation in the association between the ornamentation index (OI) and latitudes and water depths, no statistically significant linear trends, either positive or negative, were found (all *p*‐values of the Cochran–Armitage test are larger than .05; Figures 2 and 3). Moreover, the lack of a persistent and consistent trend gradient in shell ornament, irrespective of latitude or bathymetry, prevails even if the dataset was analyzed at different latitudinal (10° and 30° latitudinal bins) and bathymetrical scales (Figures 2 and 3), or using standardized residuals of the chi‐square test (Table 3). However, two somewhat mooted features need to be noted here. First, lower OI values tend to be more frequently found with higher latitudes and deeper water environments (latitudes higher than 60° or water depth deeper than 1000 m, Figures 2 and 3). Second, the differences in where the peak OI is located between the two hemispheres are also of interest: in the Northern hemisphere, the peak of OI occurs near the equator (10°–20°), whereas in the Southern hemisphere, the peak OI is situated in the mid‐latitude 30°–40° (Figure 2a,b).

Additionally, in Figure 4, regarding the influence of the distance from the continental shelf, most OI values of the cells in the coastal areas or close to the continental shelf have higher values (red and orange colors). By contrast, the cells in the pelagic areas farther away from the continental shelf usually have lower OI (white and light blue colors). At last, in most of the bioprovinces of living brachiopods proposed by Ye et al. (2021), most brachiopod communities are characterized by smooth shells, or shells with low OI, except those from bioprovinces C1, C2, ASP, Indian Ocean, and Atlantic Ocean (Figure 5).

Concerning specific ornamentation types [(*S*), (RO), (CO), (SO), and (*M*)], overall, there is no pronounced latitudinal trend gradient in terms of the distribution of standardized residuals (Table 2). Higher latitudinal bin profiles (>60°) all show a positive association with (*S*) and a negative association with (*O*) and (RO). Meanwhile, at latitude 40°–60° of both hemispheres, the standardized residuals of profiles always have a small number of absolute values (≤0.5) with (*S*) and (*O*). The latitudinal bin profiles at the latitude <40° have a very complex pattern regarding their ornamentation type.

Regarding the (RO), which is the most popular ornament type within the group (*O*), our results suggest a significant gradient trend of (ROI) present in the Northern hemisphere (Figure 6, *p*‐value <.001 for both dorsal valve and ventral valve, respectively). It means that the brachiopods at high latitudes have more radial ornamentation per width than those in lower latitudes. In other words, individual radial ornamentation becomes narrower toward higher latitudes correspondingly. By contrast, the latitudinal (ROI) curve fluctuates in the Southern hemisphere, and no significant trend in (ROI) is detected.

Finally, the positive global Moran's I values (and the positive corresponding normalized *z*‐scores) for all distance increments indicate the existence of a spatial clustering phenomenon on the shell ornamentation of living brachiopods (Figure S1). Moreover, the corresponding *p*‐values of the Spatial autocorrelation analyses are always <.05 from the beginning of the distance increment (250 km). Thus, the spatial clustering phenomenon reflected by the positive values of Moran's I is not likely caused by randomness.

## 4 DISCUSSION

### 4.1 Weak association between shell ornamentation and latitudes/bathymetry

Overall, although the shell ornamentation of living brachiopods varies markedly among different latitudes and water depths, none of the shell ornament index (OI) changes linearly along the latitude or water depth as would be predicted under a strong latitude‐predation or depth‐predation pressure scenario (*p*‐values of corresponding trend tests always ≥ .05). Nevertheless, Figures 2c,d, 3, and 4 still demonstrate an overall weak negative association between the OI values and depth. This finding, though statistically insignificant, is consistent with previous observations of living brachiopods (Zezina, 1976, 1985), fossil brachiopods (Dietl & Kelley, 2001; Leighton, 1999; Wu et al., 2019), as well as with other marine calcifiers (e.g., Mollusks; Sato‐Okoshi et al., 2010; Vermeij, 1987). However, it should be noted that our analysis of the OI changes was conducted based on a smaller latitudinal scale (Figure 2a,b) and from separate ocean basins and coastlines (Figure 7), but yet, they all show similar fluctuation profiles, with a notable absence of a strong linear trend.

A variety of factors may affect the shell ornamentation complexity of brachiopods. Among the potential drivers, the latitudinal predation pressure gradient, which postulates a negative correlation between latitudes and ornamentation complexity (Dietl & Kelley, 2001; Leighton, 1999; Wu et al., 2019), has been considered as perhaps the most important determinant, which itself is also strongly influenced by the global latitudinal temperature gradient (Reynolds et al., 2018). Dietl and Kelley (2001) proposed that a more enhanced latitudinal ornamentation gradient could be expected when the strength of the latitudinal temperature gradient is pronounced. If so, we should expect a strong linear latitudinal ornamentation gradient for living brachiopods because the time interval since the Pliocene is Earth's most recent geological interval that is characterized by the steepest latitudinal gradient of temperature since the Late Cretaceous (Zhang et al., 2019). However, no such steep latitudinal gradient of OI was detected in the present study (Figures 2 and 6), in contrast to fossil brachiopods where a negative latitudinal gradient of shell ornamentation did exist, at least for those periods that have been studied (e.g., Wu et al., 2019). Though it is unclear why such contrast exists, one observation is unequivocal: fossil brachiopods, especially those from the Paleozoic Era, had a much greater ornamentation diversity (Harper & Moran, 1997; Wu et al., 2019). For example, as one of the most effective shell ornamentations against predators (Willman, 2007), spinose representatives in the Paleozoic can be up to more than 40% at low‐ or mid‐latitudes (Dietl & Kelley, 2001), contrasting with only 2.6% of their living counterparts.

Theoretically, shell ornamentation is also known to serve a range of ecological functions other than uniquely as an anti‐predatory mechanism. A general model of consensus shows that different shell morphology of brachiopods supports different lifestyles in different habitats (Harper & Moran, 1997). For example, some productid brachiopods use spines as stabilizers on the soft substrate (Leighton, 2000; Stanley, 2020). Experimental evidence also indicates that spinose ornament can increase the resistance to transportation in a higher hydrodynamical current (Alexander, 1984; Dievert et al., 2021; Garcia et al., 2018). Nevertheless, unlike their fossil counterparts, living brachiopods have very limited life strategies. Apart from lingulides that are infaunal, other living brachiopods are all epifaunal. They can attach themselves to a substrate by a pedicle or cemented to variable but usually hard objects or substrates (Emig, 1997; Thayer, 1981). Thus, there may be a relationship between shell ornamentation and the type of substrate in which living brachiopods inhabit. However, the scarcity of information concerning the substrates of living brachiopods makes it difficult to quantitatively assess how the shell ornamentation of living brachiopods might have been influenced by the variety of substrates in a latitudinal or bathymetrical context.

The latitudinal/bathymetrical fluctuation of living brachiopods' OI should also reflect the habitats they live in today. According to field observations, most of the living brachiopods are inhabitants of relatively deep waters, cold polar regions, or cryptic habitats (e.g., submarine caves, crevices, overhangs on rock walls) where food resources and predators are limited (Bitner & Gerovasileiou, 2021; Peck, 2001; Toma et al., 2022; Zezina, 2008; Zuschin & Mayrhofer, 2009). This would mean that biotic interactions and especially predation activities would be limited in these cryptic “refuge‐type” habitats (Harper, 2022).

In addition to predation pressure potentially being a significant driver for shell ornamentation, the chemistry of marine waters could also influence shell ornamentation (e.g., ocean acidification, Khanna et al., 2013; Queirós et al., 2015), which can add more complications to the potential gradient of OI along the latitude or water depth. For example, ocean acidification can weaken the shell architecture and ornament development of marine calcifying organisms under decreasing pH and lower calcium/aragonite saturation state (Ω) (Barclay et al., 2020; Gazeau et al., 2013; Mollica et al., 2018; Waldbusser et al., 2016). However, the latitudinal OI pattern revealed in this study is inconsistent with the latitudinal surface ocean curve of [CO_3_^2^‐] (Orr et al., 2005), which has a stable high values plateau in the tropics, followed by a steep declining trend toward polar regions over 30° N/S latitudes. Similarly, a mild OI trend along the water depth cannot reflect the vertical profile of Ω with a very abrupt change in the depths ≤1000 m interval (calcite, aragonite: Zeebe & Wolf‐Gladrow, 2001). It is thus difficult to infer a robust correlation between shell ornamentation and latitudinal/bathymetrical changes with respect to seawater pH. Extending this interpretation, our study would suggest that changes in ocean pH or Ω are likely only to have a limited impact on the shell construction of living brachiopods (Cross et al., 2015, 2016; Ye et al., 2019).

Lastly, we must also acknowledge the possible effect of autocorrelation on the latitudinal/bathymetrical patterns of shell ornamentation of living brachiopods (either spatial or phylogenetic). Intuitively, this is plausible, and recent studies have already addressed such an effect on the spatial patterns of species richness (e.g., Astorga et al., 2003; Gaspard et al., 2019), and on spatial variation of shell features (Malvé et al., 2018; Marko, 2005). Regarding spatial autocorrelation, the species compositions from closer distances are usually similar, and such an effect can be varied at different scales (Gaspard et al., 2019). Therefore, spatial autocorrelation can potentially cause spatial clustering or associations of entities (or values) simply because of their spatial proximity (Dale & Fortin, 2002; Gratton et al., 2017). Our spatial autocorrelation results have uncovered such a clustering pattern of OI for living brachiopods, i.e., areas with similar OI values tend to represent areas closer to one another latitudinally (Figure S1). Usually, spatial autocorrelation or spatially nonindependent data can cause inflated type I error rates in statistical analysis (Kühn, 2007; Legendre et al., 2002). However, our *p*‐value adjustment test results consistently demonstrate the nonsignificant linear correlations between OI and latitude (Figures 2, 3 and 6). Moreover, a weak negative latitudinal gradient of OI is consistently found even when different latitudinal scales were analyzed (10° and 30°; global vs. regional coastline; Figures 2 and 6), suggesting only a minor (if any) role played by spatial autocorrelation. This reasoning is based on the notion that if spatial autocorrelation were the leading cause of a weak latitudinal ornamentation gradient of living brachiopods, a similar weak latitudinal ornamentation gradient should be expected for their fossil counterparts, but this is demonstrably not the case from previous literature (Dietl & Kelley, 2001; Leighton, 1999; Wu et al., 2019). Notwithstanding this, further statistical studies in the future are necessary for teasing out the impact of spatial autocorrelation on the formation of spatial patterns of living brachiopods. On the contrary, it also must be noted that there is no consensus among ecologists concerning the effect of spatial autocorrelation on latitudinal trends of any sort, and that appropriate statistical approaches are still being developed to detect and quantify such effects reliably (Qian et al., 2013; Willig et al., 2003).

### 4.2 Why there are proportionately so many smooth living brachiopods today?

As alluded to above, living brachiopods have two outstanding features in terms of shell ornamentation when compared to their fossil counterparts, especially Paleozoic brachiopods: low ornamentation diversity and a disproportionately high percentage of living brachiopods (nearly 60%) bearing no external ornaments except for weak growth lines (Dietl & Kelley, 2001; Leighton, 1999; Williams et al., 2000; Wu et al., 2019; Zhang & He, 2008). In particular, external spines were prevalent on Paleozoic productid brachiopod shells (in our estimate, >30% of Devonian to Permian brachiopod species had external spines, which are widely accepted as a mechanism against predatory attacks, Palmer, 1979; Leighton, 2000, 2003). However, by contrast, only approximately 2.6% of living brachiopods have spinose shells, and no long spines have been reported from living brachiopods. For Mesozoic brachiopods, the proportion of brachiopod shells with very weak ornamentation (smooth and capillate) has never been higher than 40% (Vörös, 2010).

It is difficult to interpret such a dramatic reduction in the proportion and diversity of shell ornamentation among living brachiopods when compared to their fossil counterparts. Certainly, this is unlikely the consequence of any significant reduction in predation pressure in the modern oceans. Multiple predatory marine taxa, including fish, asteroids, and gastropods, can be potential predators of brachiopods (Emig, 1997; James et al., 1992; Peck, 1993; Richardson, 1997; Witman & Cooper, 1983). However, no conclusive evidence has indicated a significant decline trend of these predators since the Cenozoic, either in diversity or abundance. In effect, the opposite is more consistent with the recent geological history in that both predation pressure and anti‐predator selection have increased and intensified since the Cenozoic (Baumiller & Gahn, 2004; Dietl & Kelley, 2001; Thompson, 1999; Vermeij, 1977, 1983).

Therefore, a paradox seems to exist concerning the relatively low‐level manifestation of ornamentation complexity of living brachiopods in an otherwise highly engaged predator–prey global marine ecosystem. This decoupling demands an alternative interpretation to the classic latitudinally mediated predation pressure theory. In our view, a more reasonable potential explanation might be that living brachiopods are not the preferred food source for many predators because of their relatively low predatory return and very limited internal flesh tissues (Peck, 1993, 2001). By contrast, bivalves have more than three times higher ash‐free dry weight compared with similar‐sized brachiopods, and the difference in nutritional return can be up to 10 times (Peck, 1993; Thayer, 1985), this might explain why living bivalves have a high diversity of shell ornamentation styles (Harper & Skelton, 1993; Klompmaker & Kelley, 2015; Ubukata, 2005).

Shell ornamentation may not be the only type of protection against predation; some living brachiopods are known to produce toxicity as a possible mechanism to deter predators (Thayer, 1981, 1985; Thayer & Allmon, 1991). Whilst this scenario may explain why certain living brachiopods have subdued shell ornamentation in order to produce toxins as a trade‐off between the two alternative self‐preservation mechanisms (Harper, 2022). The extent of toxicity acting as an anti‐predation mechanism among living brachiopod is unknown, and thus, its ability to explain the negative, albeit weak, association between OI values and latitudes, and water depths must await further studies.

Still, other less explored biological traits may also function as effective anti‐predation mechanisms as alternatives to shell ornament (Brett & Walker, 2002). In the case of brachiopods, such other traits may include shell size, thickness, and shape (Bertness, 1982; Dietl & Herbert, 2005; Melatunan et al., 2013; Peng et al., 2007; Stallings et al., 2021; Vermeij, 1987). However, no such data are currently available to elucidate these relationships for living brachiopods, and therefore, no more can be said about these factors. On the contrary, brachiopod's ability to self‐repair damage could offer a potential explanation for some brachiopods with no or reduced ornamentation. For example, recent data on living brachiopods has revealed the association between predator pressure and shell repair frequency (Harper & Peck, 2016), a finding consistent with our result in that our peak OI values at temperate latitudes (Figure 2b) closely match the highest shell repair frequency in mid‐latitudes of the Southern Hemisphere (around 40°–50°, figure 4a from Harper & Peck, 2016).

Finally, according to the escalation theory proposed by Vermeij (1987), species that fail to withstand higher predation are either more prone to extinction or restricted to certain cryptic habitats or refugia where predation pressure is low. Using this theory and accounting for the fact that many living brachiopods today are living in cryptic habitats (Peck, 2001; Thayer, 1981), it may be plausible that most living brachiopods have smooth or weakly ornamented shells, at least in part, because of their lifestyle and preference for cryptic habitats where predator pressure is low, allowing brachiopods them to enact the predator avoidance strategy rather than having to manufacture robust shell ornamentation in order to survive predation hazards (Brodie et al., 1991; Harper & Skelton, 1993). Additionally, based on the energy budget theory (Harper, 2022; Kooijman & Kooijman, 2010), cryptic habitats are usually resources‐limited and are thus only suitable for organisms with relatively low metabolism and low energy requirement (such as brachiopods), neither of which is conducive to the formation of complex shell ornamentation (Zhang & He, 2008). In other words, for brachiopods living in cryptic habitats, an energy budget compromise could have weakened their shell ornamentation development.

## 5 CONCLUSION

To sum up, living brachiopods' shell ornamentation index OI can vary at different latitudinal and bathymetrical intervals. However, no statistically significant linear associations were found between OI and latitude or OI and water depth. Also significantly, ~59% of living brachiopod species were found to be smooth except for weak growth lines. Both findings are in sharp contrast to the patterns of fossil brachiopods, which are known to exhibit a much greater ornamentation diversity and, at least for the geological periods that have been studied, a linear negative latitudinal gradient of ornamentation complexity. The reasons why living brachiopods have such a high proportion of smooth or weakly ornamented shells and fail to demonstrate an unequivocal linear latitudinal ornamentation gradient are not entirely clear but seem to be well explained by their low metabolism lifestyle and preference for cryptic (refuge‐type) habitats (e.g., deep waters, cold polar regions, fjords, and submarine rock caves) where predation pressure is low and food resources‐limited, allowing brachiopods to enact the predator avoidance strategy rather than having to manufacture robust shell ornamentation to survive in an otherwise highly engaged predator–prey global marine ecosystem.

## AUTHOR CONTRIBUTIONS

**Facheng Ye:** Conceptualization (equal); data curation (equal); formal analysis (lead); investigation (equal); methodology (lead); project administration (equal); resources (equal); software (equal); validation (equal); visualization (equal); writing – original draft (equal); writing – review and editing (equal). **Maria Aleksandra Bitner:** Data curation (equal); investigation (equal); resources (equal); validation (equal); writing – review and editing (equal). **G. R. Shi:** Conceptualization (lead); investigation (equal); methodology (equal); project administration (lead); resources (lead); supervision (lead); validation (equal); writing – review and editing (equal).

## CONFLICT OF INTEREST STATEMENT

The authors declare no conflict of interest.

### OPEN RESEARCH BADGES

This article has earned an Open Data badge for making publicly available the digitally‐shareable data necessary to reproduce the reported results. The data is available at Open data.

## Supporting information


Figure S1

## Data Availability

Data are available in the Dryad Digital Repository https://datadryad.org/stash/share/d6MThGOaT59vHRWA3tzSnXFAJxj8__dEOuaX‐MKKDeI
