# Supplementary material for: Variation of shell ornamentation with latitude and water depth—A case study using living brachiopods
Source: Ecol Evol. 2023 Apr 21;13(4):e10006. doi: 10.1002/ece3.10006 (PMC10121232; doi:10.1002/ece3.10006)
Supplement: Supplementary file 1 — Figure S1 [file ECE3-13-e10006-s001.docx]

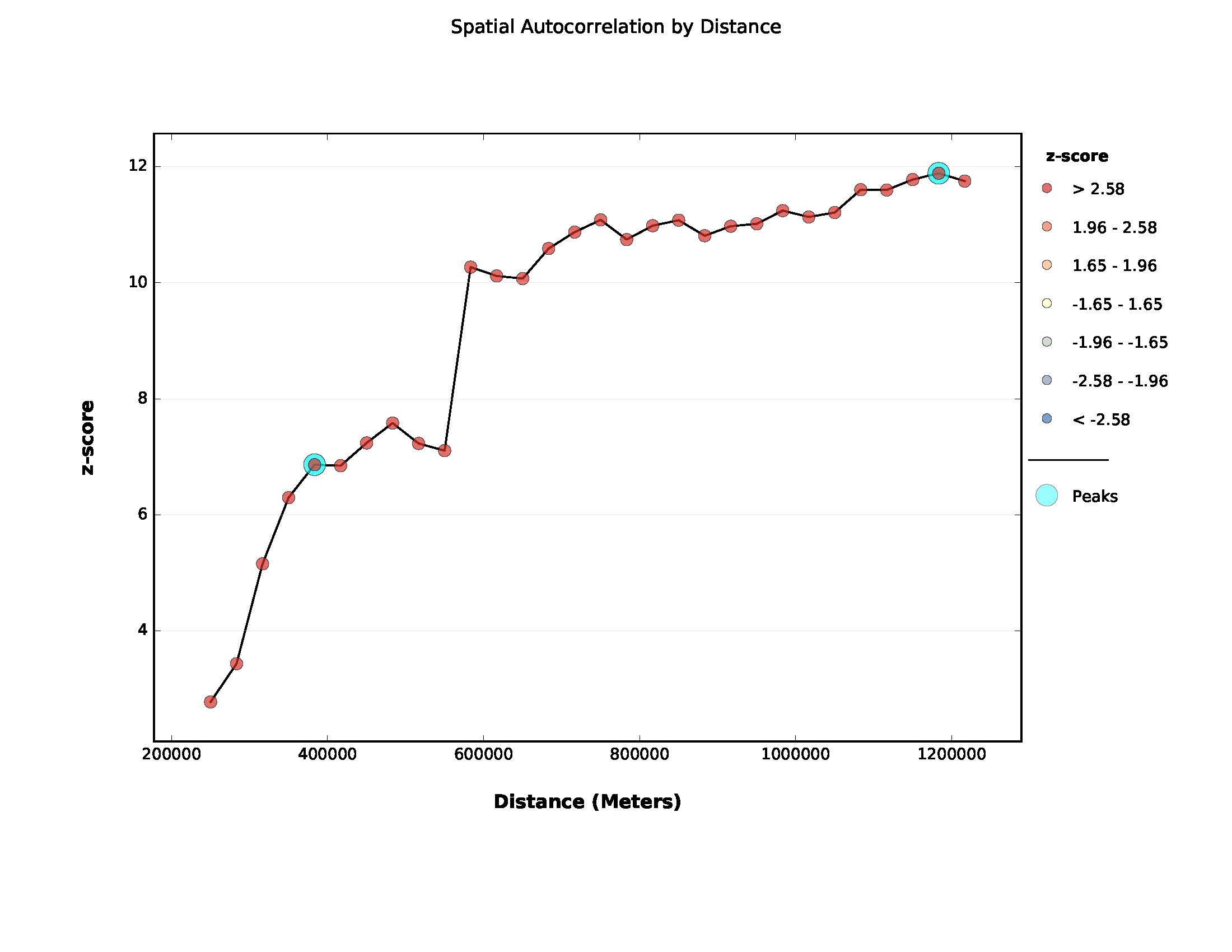


Figure S1 Results of the incremental spatial autocorrelation analysis performed used Euclidean as the distance method through ArcMap. The graph shows the corresponding z-score for each distance increment. Filled circles represent significant z-score values (*p* < 0.05). First peak distance value: 383339 m (z-score 6.865); Max peak distance value: 1183352 m (z-score 11.887).
